# Saproxylic beetles' morphological traits and higher trophic guilds indicate boreal forest naturalness

**DOI:** 10.1002/ece3.10739

**Published:** 2023-12-13

**Authors:** Ross Wetherbee, Tone Birkemoe, Ryan C. Burner, Anne Sverdrup‐Thygeson

**Affiliations:** ^1^ Department of Environmental Sciences Western Norway University of Applied Sciences Sogndal Norway; ^2^ Faculty of Environmental Sciences and Natural Resource Management Norwegian University of Life Sciences Ås Norway; ^3^ U.S. Geological Survey, Upper Midwest Environmental Sciences Center La Crosse Wisconsin USA

**Keywords:** biodiversity monitoring, community composition, forest management, functional traits, saproxylic beetles, species richness

## Abstract

Forests contribute to numerous ecosystem functions and services and contain a large proportion of terrestrial biodiversity, but they are being negatively impaced by anthropogenic activities. Forests that have never been clear‐cut and have old growth characteristics, termed “near‐natural,” often harbor different and richer species assemblages than managed forests. Alternative management strategies may be able to balance the needs of biodiversity with the demands of forestry, but evaluation efforts are limited by the challenges of measuring biodiversity. Species richness is frequently used as a simple measure of biodiversity, but research indicates that it may not adequately capture community‐level changes. Alternatively, trait‐based measures of biodiversity may prove to be useful, but research is lacking. In this paper, we use a large dataset that includes 339 obligate saproxylic beetle species collected over a decade in the boreal region throughout southern Norway to: (1) establish if there is a difference in beetle community composition between near‐natural and managed forests; and (2) determine which measures of beetle biodiversity best indicate forest naturalness. We arranged the sites in an ordination space and tested for differences in community composition between these forest types. We also tested different measures of biodiversity to determine which were the most predictive of forest naturalness. We found a clear difference in community composition between near‐natural and managed forests. Additionally, three measures of biodiversity were most predictive of forest naturalness: proportional abundance of predators, community weighted mean (CWM) of wing length, and CWM of body roundness. The probability that a forest was near‐natural increased with the proportional abundance of predators but decreased with CWM wing length and body roundness. Although species richness was higher in near‐natural forests, the effect was not significant. Overall, our findings underscore the conservation value of near‐natural forests and highlight the potential of several measures of biodiversity for determining forest quality.

## Graphical abstract

There was a clear difference in saproxylic beetle community composition between near‐natural and managed forest, and three measures of saproxylic beetle biodiversity were most predictive of forest naturalness: proportional abundance of predators, CWM of wing length and CWM of body roundness. Although species richness was higher in near‐natural forest, the effect was not significant. Overall, our findings underscore the conservation value of near‐natural forests and highlight the potential of these measures of biodiversity for determining forest quality.

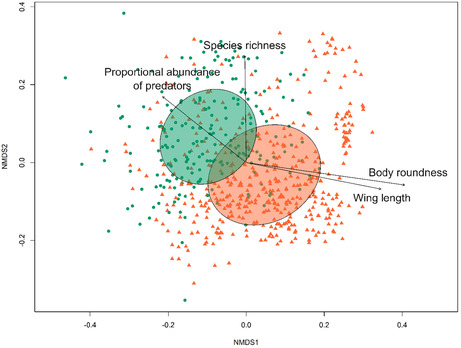

## 1 INTRODUCTION

Human societies are deeply dependent on forests, both for economic gains and for our overall wellbeing (IPBES, 2022). Forests contribute to numerous critical ecosystem functions and services, such as the regulation of the climate, the absorption of large amounts of carbon from the atmosphere, and the provisioning and regulation of fresh water. Additionally, forests contain a large proportion of total terrestrial biodiversity (Oettel & Lapin, 2021). This is especially important because there has been a dramatic loss of global biodiversity in the last 50 years, and this decline is predicted to continue or accelerate unless there is rapid, transformative change (Díaz et al., 2019; IPBES, 2018; Reid et al., 2005). Large‐scale declines in forest biodiversity may compromise many of the ecosystem functions and services on which humans rely (Brockerhoff et al., 2017).

Boreal forests represent the single largest pool of living biomass on Earth and span extensive areas across Earth's northern regions (DeAngelis, 2008). These forests play a crucial role as major providers of ecosystem services, such as carbon storage and clean water, and serve as habitats for globally significant wildlife populations (Frelich, 2020). Although there are large areas of unlogged primeval forests, boreal forests are nevertheless threatened by unregulated logging, mining, oil extraction, and climate change (Frelich, 2020). In Northern Europe, intensive forestry (especially the extensive use of clear cutting) has been identified as a main threat to boreal forest biodiversity (Kuuluvainen, 2009). Intensive forestry is detrimental to biodiversity, primarily because it homogenizes forest structure by reducing the number of tree species, tree age classes, and the amount and diversity of dead wood (Bütler et al., 2013; McGeoch et al., 2007; Oettel & Lapin, 2021). Forests that have never been clear‐cut and that have old growth characteristics such as age class heterogeneity and a high diversity and amount of dead wood are often termed “near‐natural” (Jacobsen et al., 2020; Paillet et al., 2010). Forests with these old growth characteristics have been found to harbor different and richer species assemblages than managed forests (Jacobsen et al., 2020; Martikainen et al., 2000; Similä et al., 2002). Consequently, the presence of near‐natural forests increases the regional species pool and has a positive influence on the composition and richness of species found in adjacent, managed forests (Butaye et al., 2002).

Managing forests sustainably is challenging for many reasons (Kuuluvainen et al., 2019), and good decision‐making can only be accomplished based on sound science. Unfortunately, measuring and monitoring forest biodiversity across space and time has proven to be a major challenge in and of itself (Burrascano et al., 2021). This challenge arises due to the complex nature of forests, which harbor large amounts of biodiversity with taxon‐specific responses that include seasonal appearances and substantial inter‐annual variability, along with processes unfolding across large spatial and temporal scales (Burrascano et al., 2021; Storch et al., 2023). Therefore, trends in species richness may be insufficient to capture more complex changes in biodiversity in response to human impacts and a rapidly changing climate (Hillebrand et al., 2018). It has been suggested that focusing on forest structure, rather than directly on biodiversity, might be one solution to this problem (Storch et al., 2023). However, it remains unclear to what extent forest structure or other simple metrics of biodiversity can act as a proxy for forest biodiversity as a whole.

One way to make monitoring more feasible is to focus on specific species or groups of species that are characteristic of habitats and which indicate the condition of the community at large (Gao et al., 2015). These species or groups of species are termed biodiversity indicators, and they are a frequently used tool to monitor the status of biodiversity, changes in biodiversity, and the effects of management actions (Oettel & Lapin, 2021). One group of species that may indicate forest conditions are saproxylic insects (i.e., species that are dependent on dead wood for all or part of their life cycle) (Stokland et al., 2012). Saproxylic beetles are a major component of forest biodiversity and play important roles in several ecosystem processes (Stokland et al., 2012; Ulyshen, 2013, 2016; Wetherbee, Birkemoe, Skarpaas, et al., 2020; Wetherbee, Birkemoe, & Sverdrup‐Thygeson, 2020). Also, there is considerable evidence that their communities respond to old‐growth forest characteristics (Dodelin, 2010; Jacobsen et al., 2020; McGeoch et al., 2007; Paillet et al., 2010; Seibold et al., 2019; Similä et al., 2002). Thus, saproxylic beetle communities may be good biodiversity indicators (Gao et al., 2015; Stokland et al., 2012).

Much of the work using saproxylic beetles as biodiversity indicators for forest management has focused on species richness, which likely overlooks other impacts of management (Fleishman et al., 2006). For example, a reduction in forest structure due to intensive management has been found to alter insect community composition (Jacobsen et al., 2020; Leidinger et al., 2019; McGeoch et al., 2007; Similä et al., 2002), impact specialized species and higher trophic levels (Cagnolo et al., 2009; Komonen et al., 2000; Laaksonen et al., 2020; Pilskog et al., 2016), and reduce the functional diversity of the community (Drag et al., 2023; Murry et al., 2017; Neff et al., 2022; Staab et al., 2023). Few of these responses would be captured by a simple measure of species richness. Yet, the results from an analysis of community composition do not provide a generalizable understanding of how the community changes or the potential consequences of these changes for ecosystem functions and services, and thus have limited uses as biodiversity indicators. Trait‐based approaches, on the other hand, may provide useful indicators of biodiversity, as they can detect subtle shifts in communities in ways that provide a more generalizable and mechanistic understanding of ecosystem functioning and assembly processes (Burner et al., 2022; de Bello et al., 2021). Functional traits, defined as a phenotypic aspect of an organism's morphology, physiology, phenology, or behavior that affects the organism's fitness or influences an ecosystem process (de Bello et al., 2021; Violle et al., 2007), can be especially useful in this effort. But despite the advantages of trait‐based approaches, there has been a limited amount of research relating trends in insect functional traits to forest management (Murry et al., 2017).

In this paper, we compare saproxylic beetle assemblages collected in near‐natural and managed forests within the boreal region of southern Norway. We use a large data set that includes 339 obligate saproxylic beetle species collected over a decade across 270 sites throughout southern Norway. The aims of the study were first to establish if there is a difference in saproxylic beetle community composition between near‐natural and managed forests, and then to determine which measures of beetle biodiversity best capture the differences in forest naturalness. We considered classical taxonomic‐based measures such as species richness and Shannon's diversity index, as well as measures based on species' functional traits. This method enables a broader application to other ecosystems, where comparable traits or community structures could act as indicators of the overall “condition” of the community. Deciphering this information solely from the habitat can be challenging, especially when the management history is less well known and considering the slow pace of habitat changes. Thus, by identifying which aspects of saproxylic beetle diversity are the most indicative of forest naturalness, we may be able to identify biodiversity indicators that can be used to assess forest conditions in this and other systems.

## 2 MATERIALS AND METHODS

**FIGURE 1:**
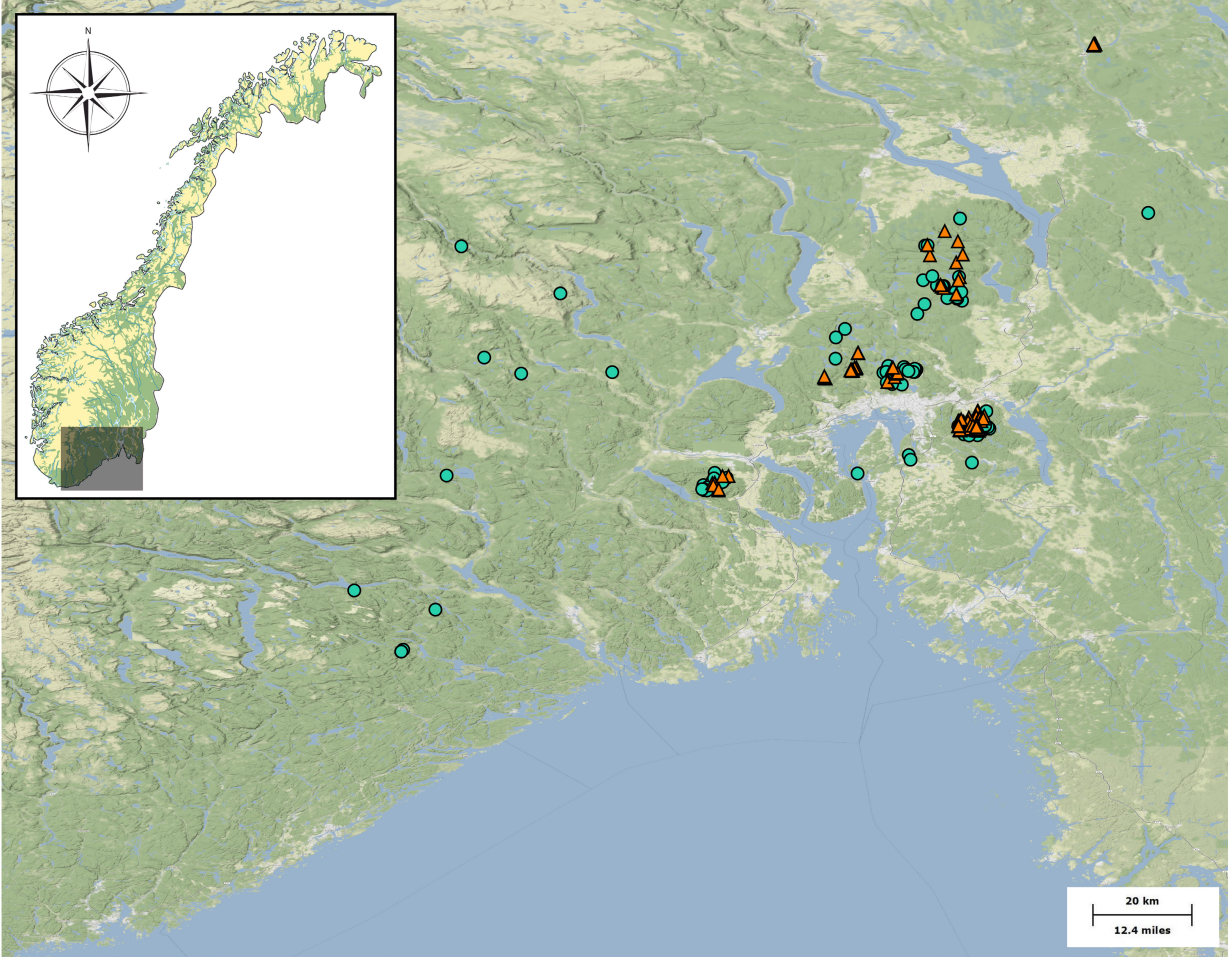
Map of beetle sampling sites in southern Norway. Sites were classified as either near‐natural (green circles, *N* = 97) or managed forest (orange triangles, *N* = 172). Near‐natural forests were defined as forests that have never been clear‐cut and that have older trees, higher dead wood volume, and higher heterogeneity in forest structure compared to managed forests. Managed forests were managed as sustainable production forests within the guidelines of the Program for the Endorsement of Forest Certification schemes (PEFC) and were in closed canopy forests that have been intensively managed and clear‐cut in the past.

The data were compiled from previous work (Birkemoe & Sverdrup‐Thygeson, 2015; Burner et al., 2020; Fossestøl & Sverdrup‐Thygeson, 2009; Sverdrup‐Thygeson et al., 2017; Sverdrup‐Thygeson & Ims, 2002; Vindstad et al., 2020), where beetle communities were sampled at 270 sites (near‐natural = 98, managed = 172) intermittently between 1997 and 2007 in the boreal region in southern Norway (Figure 1) as part of eight sampling projects. Most of the Norwegian forest in productive areas has experienced some form of management. However, the dominant forest management model in Norway shifted from selective harvesting to stand replacement around 1950 (Helseth et al., 2022). Currently, the mean stand age of production forest in Norway is approximately 70 years, with 27% being 40 years or younger, 41% between 41 and 100 years, and 32% being older than 100 years (Breidenbach et al., 2020). As is typical of boreal regions, the forests included in our study were dominated by conifers (*Pinus sylvestris* and *Picea abies*), with some deciduous tree species, and were classified as being either near‐natural or managed based on their management history, as in previous research (Burner et al., 2021; Jacobsen et al., 2020). Near‐natural forests were defined as forests that have never been clear‐cut and contain older trees, higher dead wood volume, and higher heterogeneity in forest structure compared to managed forests (Storaunet et al., 2005). Sites classified as near‐natural forest were located in nature reserves, woodland key habitats, or in areas about to receive such status (Burner et al., 2021; Jacobsen et al., 2020). Managed forests were managed as production forests within the regulations of the PEFC (the Program for the Endorsement of Forest Certification Schemes, Norway, pefc.org). Lastly, only forests of mature age were included in the dataset, and all clear‐cuts were excluded because research indicates that clear‐cut sites are inherently different from sites with mature trees (Burner et al., 2021; Jacobsen et al., 2020).

In all projects, beetles were sampled using flight intercept traps that were either hanging in a focal tree, Norway spruce (*P. abies*) or Eurasian aspen (*Populus tremula*), or were free hanging in the forests. Trap substrates (focal tree or free hanging) were roughly comparable between forest management categories (Table S1). The design of the flight intercept trap was generally the same, consisting of two clear, intersecting plastic barriers above a funnel that leads to a collection vile. However, one sampling project used Polish IBL‐2 traps, which consist of a triangular single‐plane mesh barrier above a funnel that leads to a collection vial (*n* = 38 sites). We controlled for this and other sources of introduced variation by including the sampling project as a random effect in our models (refer to section on statistical analysis for more details). Sampling took place from May to August, and traps were emptied once a month during that time. All beetles collected at a single site were pooled for each year for the analysis. Beetles were identified to species level based on their morphology, following the taxonomy of the Norwegian Biodiversity Information Centre (NBIC, 2018), and categorized as obligative saproxylic according to the Saproxylic Database compiled by Dahlberg and Stokland (2004). Only obligate saproxylic species were included in our analyses. For additional details related to beetle sampling, please refer to the original research (Birkemoe & Sverdrup‐Thygeson, 2015; Fossestøl & Sverdrup‐Thygeson, 2009; Sverdrup‐Thygeson et al., 2017; Sverdrup‐Thygeson & Ims, 2002; Vindstad et al., 2020).

For measures of saproxylic beetle biodiversity, we included both classical taxonomic measures (e.g., species richness) as well as trait measures (Table 1). Choosing which traits to focus on is a major challenge in trait‐based ecology (de Bello et al., 2021). We therefore chose to include the six morphological traits identified by Hagge et al. (2021) as being most associated with saproxylic beetle extinction risk: body length, body width, body roundness, wing length, wing load, and mandibular aspect. These traits were corrected for body size by extracting the residuals from a phylogenetically corrected regression model that predicted the respective trait with body size (Hagge et al., 2021). We also included data from Wetherbee, Birkemoe, Skarpaas, et al., 2020; Wetherbee, Birkemoe, & Sverdrup‐Thygeson, 2020 regarding whether the species was a predator or a decomposer. Species were assigned to the groups based on published literature regarding both adult and larval diets, and therefore the groups were not mutually exclusive (Wetherbee, Birkemoe, Skarpaas, et al., 2020; Wetherbee, Birkemoe, & Sverdrup‐Thygeson, 2020).

**TABLE 1:** Measures of saproxylic beetle biodiversity and the correlation between each of these measures and the ordination axes (*R* ^2^).

Measure of biodiversity	Type	NMDS *R* ^2^
CWM body roundness	Trait	.555
CWM wing length	Trait	.414
Shannon diversity index	Taxonomic	.278
Prop. abundance of predators	Trait	.271
Species richness	Taxonomic	.267
FDis	Trait	.264
CWM body length	Trait	.253
Predator species richness	Trait/Taxonomic	.236
FEve	Trait	.208
FRic	Trait	.203
Decomposer species richness	Trait/Taxonomic	.184
Total abundance	Taxonomic	.182
Prop. abundance of decomposers	Trait	.145
CWM mandibular aspect	Trait	.103
CWM wing load	Trait	.039
CWM body width	Trait	.027

*Note*: Beetles were sampled with flight intercept traps in either near‐natural or managed forests. The *R* ^2^ value was obtained by a multiple regression model calculated by permutation in the vegan package. All variables were related to ordination axes more than by random chance (α < .003 after applying the Bonferroni correction for multiple testing).

Abbreviations: CWM, community weighted mean; FDis, functional dispersion; FEve, functional evenness; FRic, functional richness.

### 2.1 Statistical analysis

We first calculated several measures of biodiversity (Table 1) for the saproxylic beetles in the dataset (refer to Table S2 for full species list), including the taxonomic measures of species richness, Shannon diversity index, and total abundance for each sampling location. We also calculated species richness and the proportional abundance of saproxylic beetles within two functional groups: predators and decomposers. Furthermore, we computed the community weighted mean (CWM) for each of the six morphological traits. CWM represents the average trait value across all individuals in the community, weighted by their relative abundances (Garnier et al., 2004). Finally, to account for the main aspects of functional diversity, we calculated functional dispersion (FDis), functional richness (FRic), and functional evenness (FEve) using a combination of these six morphological traits. FDis is a measure of dispersion in trait space and is calculated as the mean distance of all species (weighted by abundances) to the centroid of the community in multidimensional trait space (Laliberte & Legendre, 2010). FRic quantifies the total volume of multidimensional trait space (or convex hull) occupied by the species in a community, reflecting the extent of functional differentiation and diversity among coexisting species (Villéger et al., 2008). FEve is a measure of the regularity of distance in trait space (distance to neighbor) and is connected to niche differentiation (Mason et al., 2005), with lower values indicating higher trait redundancy among species. We used the function “dbFD” from the “FD” package (Laliberte & Legendre, 2010) to calculate these measures of beetle biodiversity for each site. All analyses were completed in R version 4.3.0 (R Development Core Team, 2023).

In order to establish if there was a difference in beetle community composition between near‐natural and managed forests, we arranged the sites in ordination space with a nonmetric multidimensional scaling (NMDS) ordination based on the Bray–Curtis dissimilarity matrix (Legendre & Legendre, 2012), which was calculated from the species abundance matrix. The NMDS was carried out with the “metaMDS” function in the vegan package (Oksanen et al., 2022). The ordination was initially carried out with two dimensions and 999 iterations, but due to high stress (>2), we increased the number of dimensions to three (Figures S2 and S3). We subsequently drew ellipses around the different forest types based on the standard deviation of points (sites) within each category with the function “ordiellipse” in the vegan package (Oksanen et al., 2022). To test for a significant difference in species composition between the forest types, we used a permutational analysis of variance (PERMANOVA) (Anderson et al., 2017) with forest type as a fixed variable and the sampling project as a random variable (strata) using the function “adonis2” in the vegan package (Oksanen et al., 2022). Because this required multiple tests, we applied the Bonferroni correction to the *p* value and obtained a new critical threshold (α) of .003 (Haynes, 2013).

**FIGURE 2:**
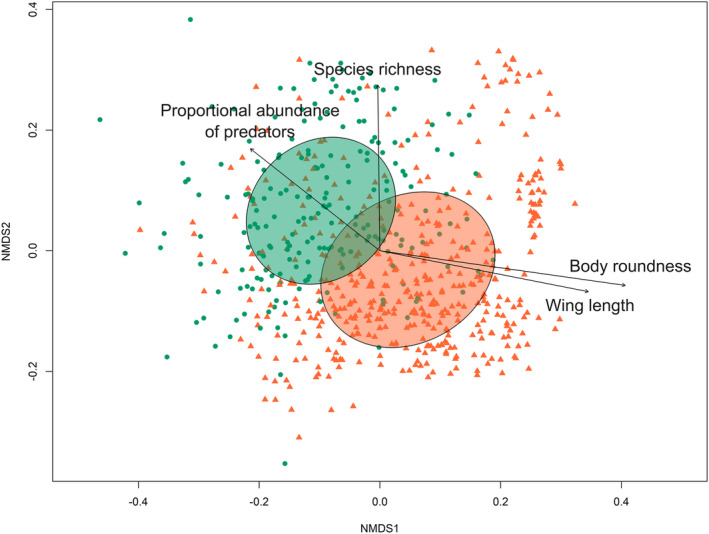
A plot of the first two dimensions of the nonmetric multidimensional scaling ordination of all study sites. Saproxylic beetles were sampled with flight intercept traps in either near‐natural (green circles) or managed forests (orange triangles). The 95% confidence ellipse for each forest type is shown, and species richness and the three biodiversity measures identified with the generalized linear mixed effect model (GLMM) as being most predictive of forest naturalness are plotted as vectors. All variables were related to ordination axes more than by random chance (alpha < .003, refer to Table 2 for model results).

To assess which measures of biodiversity best capture the differences between forest naturalness, we used the envfit function from the vegan package (Oksanen et al., 2022). This analysis allowed us to evaluate the alignment and correlation between the measures of biodiversity and the ordination axes (Table 1). We then visualized these relationships by plotting the measures of biodiversity as vectors on the ordination plot (Figure 2 and Figures S1 and S2). We subsequently built a generalized linear mixed effect model (GLMM) with binomial distribution that predicted forest naturalness (where near‐natural forests = 1 and managed forests = 0), with the biodiversity measures (Table 1) as fixed effects. Since the dataset used in the study was a combination of eight sampling projects, which included different years and slightly different geographic regions and trap types, we included the sampling project as a random effect. Models were created with the function “glmmTMB” from the package glmmTMB (Brooks et al., 2017). In addition to the biodiversity measures, we also included latitude and longitude as predictor variables in the model. All predictor variables were scaled by subtracting the mean and dividing by the standard deviation of the data matrix using the scale base function in R. To avoid multicollinearity, we calculated the variance inflation factor (VIF) for all the predictor variables and systematically dropped the variables with the highest VIF (Table S3). This was done until only variables with a VIF below three remained (Zuur et al., 2009), and these variables were used in our full model (Table 2). We subsequently made a reduced model using backwards model selection with the function “dropterm” in the MASS package (Venables & Ripley, 2002) based on the Akaike information criterion (AIC). We also made alterative single‐covariate models that predicted forest naturalness as a function of observed species richness, because this is a commonly used measure of biodiversity but was removed during our model selection process due to collinearity, and as a function of body roundness because it explained the most variation in the ordination but was also removed due to collinearity (for model results from the excluded variables, refer to Table S4). Finally, we compared these two additional models to the full and reduced models using AIC. The pseudo‐*R* ^2^ was calculated with the function “r.squaredGLMM” from the package MuMIn (Lee et al., 2013). Models were also checked for influential observations and patterns between the residuals and all potential predictor variables, sampling date, and geographic location (Zuur et al., 2009).

**TABLE 2:** Results from models that predicted forest naturalness using measures of saproxylic beetle biodiversity.

Full model	Estimate	Std. error	*p* Value	AIC	*R* ^2^m	*R* ^2^c
Intercept	111.522	77.680	.1511	377	.040	.908
CWM body length	−0.276	0.216	.202			
CWM wing length	−0.451	0.209	.031*			
CWM mandibular aspect	0.056	0.186	.762			
CWM body width	0.041	0.173	.812			
FDis	0.254	0.218	.244			
Prop. abundance of predators	0.353	0.178	.047*			
Shannon diversity index	−0.089	0.207	.666			
FEve	−1.225	2.121	.563			
Latitude	−1.967	1.370	.151			
Longitude	0.634	0.629	.313			
Reduced model						
Intercept	0.358	2.657	.893	369	.011	.915
CWM wing length	−0.490	0.193	.011*			
Prop. abundance of predators	0.327	0.151	.031*			
Alternative models						
Intercept	0.955	3.2946	.772	377	.003	.873
Species richness	0.411	0.2338	.079			
Intercept	0.527	2.877	.855	362	.011	.847
CWM body roundness	−0.726	0.173	<.001*			

*Note*: Forest naturalness (near‐natural = 1, managed = 0) was modeled with a generalized linear mixed effect model (GLMM) with binomial distribution, measures of beetle biodiversity, latitude, and longitude as fixed effects, and the sampling project as a random effect. Only variables with a variance inflation factor (VIF) below three were included in the full model, and the reduced model was obtained by performing backward model selection using the Akaike information criterion (AIC). We also made alternative models that predicted forest naturalness based on species richness and body roundness because the ordination indicated that they may be important predictors of forest naturalness, but they could not be included in the full model due to collinearity.

Abbreviations: CWM, community weighted mean; FDis, functional dispersion; FEve, functional evenness.

* indicates significance at .05 threshold.

## 3 RESULTS

Between 1997 and 2007, we collected 339 saproxylic beetle species and 70,695 individuals from 270 sites (near‐natural = 98 and managed = 172). The mean species richness per year at near‐natural sites was 44 (min = 7, max = 149), whereas the mean species richness in managed forests was 30 (min = 5, max = 100). We found a clear difference in beetle taxonomic community composition between the forest types. The results from the PERMANOVA test indicated that there was a significant difference between the forest types after 999 permutations (*R* ^2^ = .045, df = 654, *F* = 30.782, *p* = .001). Additionally, all the measures of biodiversity were significantly related to ordination axes (Table 1). Furthermore, the NMDS showed separation in ordination space between forest types along both NMDS1 and NMDS2 (stress = 0.198, Figure 2).

**FIGURE 3:**
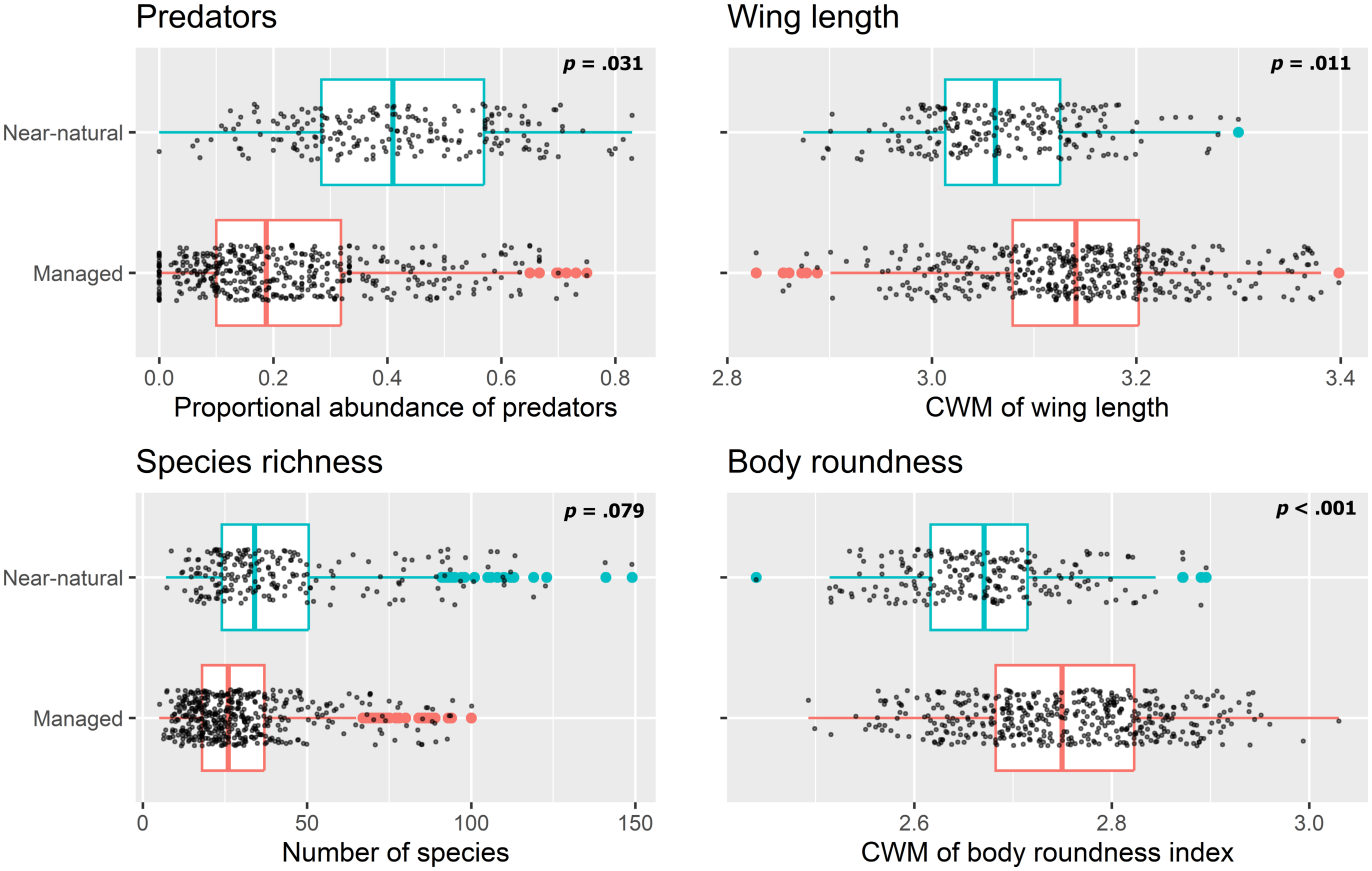
Boxplots of the three measures of saproxylic beetle biodiversity identified with the generalized linear mixed effect (GLMM) model as being most predictive of forest naturalness and species richness plotted for near‐natural and managed forests. The boxplots show the median, first, and third quartiles, with whiskers that extend 1.5 times the interquartile range. The *p* value in the top right corner of the plots is for the effects of each variable from the models that predicted forest naturalness (refer to Table 2 for models and results). CWM, community weighted mean.

**FIGURE 4:**
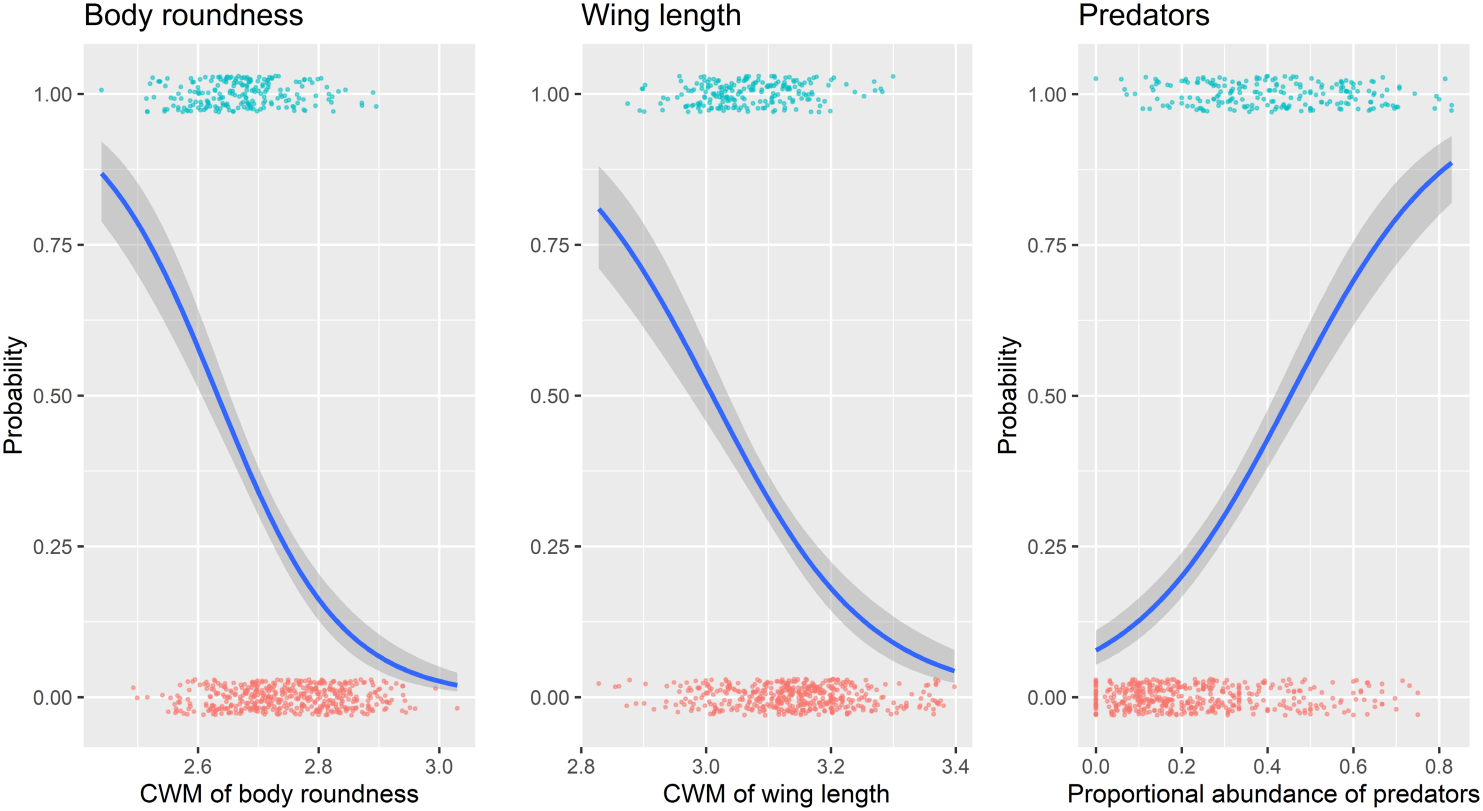
Plots of the marginal effects of each variable from the models that best predicted forest naturalness (near‐natural = 1 (green), managed = 0 (orange)) with measures of saproxylic beetle biodiversity. A model including only the community weighted mean (CWM) of body roundness community ranked highest using the Akaike information criterion (AIC), followed by a model that included the community weighted mean (CWM) of wing length and the proportional abundance of predators. The probability of the forest being near‐natural decreased with increasing body roundness (left) and wing length (middle) but increased as the proportional abundance of predators increased (refer to Table 2 for model results). Other model covariates were held at their mean across the gradients.

Overall, our analysis pointed to three measures of biodiversity that were the most predictive of forest naturalness: proportional abundance of predators, CWM of wing length, and CWM of body roundness (Table 1 and Figures 2 and 3). After the model section process, we obtained a reduced model that included both the proportional abundance of predators and the CWM of wing length as predictors (Table 2 and Figure 4). The model indicates that a forest is more likely to be near‐natural with an increasing proportional abundance of beetle predators (est. = 1.760, *p* = .023) and decreasing beetle wing length (est. = −4.614, *p* = .013). We also made two alternative models that predicted forest naturalness with body roundness and with species richness. These variables were excluded from the full model due to collinearity, but the ordination indicated that they may be important predictors of forest naturalness. Results from these models indicated that a forest is more likely to be near‐natural with decreasing body roundness of saproxylic beetles (est. = −7.386, *p* < .001) and that a forest is more likely to be near‐natural with an increasing number of species, although this effect was not significant (est. = 0.018, *p* = .079). When the AIC of the full, reduced, and two single‐covariate models were compared, the model including only body roundness was the lowest (Table 2).

## 4 DISCUSSION

This study had two main aims. First, to establish if there was a difference in saproxylic beetle community composition between near‐natural and managed forests, and second, to determine which measures of biodiversity best capture the differences in forest naturalness. We found that the species composition of saproxylic beetles differed between near‐natural and managed forests. We also found that the proportional abundance of predators, CWM of wing length, and CWM of body roundness of saproxylic beetles were the most predictive of forest naturalness. Although species richness tended to be higher in near‐natural forests, the effect was not significant. It should also be noted that these models had low predictive power, and this underscores the notable variation within saproxylic beetle communities. However, the ordination revealed similar patterns, with the highest correlation being between these same measures of biodiversity and the ordination axes. Thus, our work highlights several aspects of saproxylic beetle communities that are most sensitive to intensive forest management and contributes to the large body of knowledge indicating that anthropogenic activities alter the community composition and traits of forest‐dwelling insects (Habel et al., 2019).

The differences between near‐natural and managed forests were best captured by differences in community composition and trait metrics rather than species richness. Species richness is the primary measure of biodiversity, and previous research has found that it is influenced by forest management intensity (Habel et al., 2019; Jacobsen et al., 2020; Martikainen et al., 2000; Müller et al., 2008). However, other research indicates that the species richness of saproxylic beetles or forest beetles more broadly is not necessarily higher in natural versus managed forests, while simultaneously finding large differences in other measures of biodiversity, such as community composition (Martikainen et al., 2000; Similä et al., 2003). Therefore, a focus on species richness as the primary (or only) measure of biodiversity may lead to confusion about anthropogenic impacts on nature (Hillebrand et al., 2018). For example, some researchers have concluded that the literature remains equivocal in regards to the impacts of intensive forest management on biodiversity (as measured by species richness) and suggests that increases in management intensity may not be detrimental to forest biodiversity (Asbeck et al., 2021), despite considerable evidence to the contrary (Jacobsen et al., 2020; Kuuluvainen, 2009; Kuuluvainen et al., 2019; Martikainen et al., 2000; McGeoch et al., 2007; Miller et al., 2009; Paillet et al., 2010; Savilaakso et al., 2021). Furthermore, reductions in environmental quality can result in temporary, localized increases in species richness or abundance due to the temporal dynamics of emigration and extinction (Hillebrand et al., 2018). Thus, measurements beyond species richness are needed to better measure and monitor the effects of forest management.

Our results indicate that some traits of saproxylic beetles may be more indicative of the effects of intensive forestry and may be more useful as biodiversity indicators. We found that the proportional abundance of predators, the CWM of wing length, and the CWM of body roundness of saproxylic beetles were the best predictors of forest naturalness. Specifically, the probability that the forest was near‐natural increased with the proportional abundance of predators and with decreasing CWM of wing length and body roundness. Specialized predators on the top of food chains are especially vulnerable to habitat loss and fragmentation (With, 2019), and research focused on boreal forests has also found that habitat loss and fragmentation truncate the food chains of saproxylic species (Cagnolo et al., 2009; Komonen et al., 2000). This clearly indicates that insect predators are vulnerable to intensive forest management, a point that has been corroborated by recent research (Staab et al., 2023). Insect predation is an important ecosystem function that is sensitive to landscape simplification (Dainese et al., 2019), and greater diversity of beetle predators is related to higher insect predation rates in forests (Wetherbee, Birkemoe, & Sverdrup‐Thygeson, 2020). Also, research on the natural enemies of bark beetles indicates that invertebrate predators play a major role in regulating their populations (Khanday et al., 2018; Wegensteiner et al., 2015). Therefore, our results highlight the potential cost of intensive forest management in that it may degrade forest resilience by diminishing predatory communities, potentially increasing the chances of insect pest outbreaks (Snyder, 2019).

Our findings also indicate that species with reduced dispersal ability are sensitive to intensive forest management. Research has also found that traits related to dispersal ability, including wing length, decreased with the proportion of broadleaf trees (Neff et al., 2022). Neff et al. (2022) suggest that the mechanism behind this result is that conifers have been relatively recently introduced into Central Europe for forestry purposes, and conifer specialists with low dispersal abilities have not been able to establish themselves yet. However, this interpretation does not fit well with our results since we found similar effects in Northern Europe, where conifers have long been part of forests (Binney et al., 2017). An alternative explanation is related to the stability–dispersal hypothesis, which states that species using more stable habitat have lower dispersal ability than species using more ephemeral habitat (Southwood, 1977). Research related to forest beetles has found support for this hypothesis, in that guilds associated with stable habitat were affected by landscape structures at smaller geographical scales than guilds of less stable habitats (Percel et al., 2019). Unfortunately, stable microhabitats, such as tree hollows and large‐diameter dead wood, are rare or entirely absent in managed forests (Bütler et al., 2013). Thus, the loss of stable microhabitats due to intensive forest management presents a major conservation challenge.

Additionally, we found that the roundness of the saproxylic beetle's body was predictive of forest naturalness. Other research has found some indication that beetle body shape responds to forest structure (Barton et al., 2011; Micó et al., 2020; Hagge et al., 2021; Neff et al., 2022; Traylor et al., 2023), and this is enough to indicate that there may be an important mechanism at work. However, which mechanisms underlie this trait in relation to forest structure remain an open question. Some research indicates that body roundness increases with microhabitat openness (Barton et al., 2011; Neff et al., 2022), whereas other research indicates that it may be associated with tree hollows (Micó et al., 2020). Hagge et al. (2021) suggest that body flatness (being the opposite of roundness) may be associated with phylum feeders. None of these explanations fit well with our results since body roundness was a negative predictor of forest naturalness, and near‐natural forests are typically more open and more likely to have hollow trees than managed forests. However, recent research indicates that body roundness is negatively associated with forest age (Traylor et al., 2023), and this corresponds with our results.

Interestingly, CWM of body roundness was negatively correlated with the proportional abundance of predators and positively correlated with CWM wing length in our data set. Thus, it is difficult to know which (if any) trait is directly related to forest naturalness. It is important to point out that these are site‐level measures of functional diversity, and this does not mean that these traits are correlated for any single species. Instead, it indicates that e.g. sites with a high proportional abundance of predators or sites with a low mean value for wing length also tend to have species that are less round. Systematic differences in this body shape at the site level could have implications for how the community responds to environmental change since body shape influences species' cold and water loss tolerance (de Bello et al., 2021; Porter & Kearney, 2009). Despite the lack of a clear mechanism, body roundness was predictive of forest naturalness, is a simple trait to measure, and thus may have utility for research and monitoring programs.

## 5 CONCLUSION

In conclusion, this study provides evidence of a clear difference in saproxylic beetle community composition between near‐natural and managed boreal forests, with near‐natural forests supporting beetle assemblages with distinct trait compositions. Our results indicated that although species richness may be influenced by forest management, it is not necessarily a good predictor of forest naturalness. In contrast, the functional traits of wing length, proportional abundance of predators, and body roundness were identified as the best predictors of forest type. These results indicate that species at higher trophic levels and with reduced dispersal ability are sensitive to intensive forest management. Systemic changes in a community's trait composition and changes to higher trophic levels may have consequences for ecosystem functioning and biological responses to environmental changes. Overall, our findings underscore the importance of preserving near‐natural boreal forests for their conservation value and highlight the potential of several measures of biodiversity for determining forest quality for future monitoring and management efforts.

## AUTHOR CONTRIBUTIONS

**Ross Wetherbee:** Conceptualization (equal); data curation (lead); formal analysis (lead); investigation (equal); writing – original draft (lead). **Tone Birkemoe:** Conceptualization (equal); funding acquisition (equal); investigation (equal); methodology (equal); writing – original draft (supporting). **Ryan C. Burner:** Conceptualization (supporting); formal analysis (supporting); investigation (equal); writing – original draft (supporting). **Anne Sverdrup‐Thygeson:** Conceptualization (equal); funding acquisition (equal); investigation (equal); methodology (lead); writing – original draft (supporting).

## CONFLICT OF INTEREST STATEMENT

The authors have no conflict of interest to declare.

## Supporting information


Appendix S1

## Data Availability

Data are available on Zenodo: https://zenodo.org/record/8272489.
