# Supplementary material for: Saproxylic beetles' morphological traits and higher trophic guilds indicate boreal forest naturalness
Source: Ecol Evol. 2023 Dec 13;13(12):e10739. doi: 10.1002/ece3.10739 (PMC10716668; doi:10.1002/ece3.10739)
Supplement: Supplementary file 1 — Appendix S1 [file ECE3-13-e10739-s001.docx]

Supplementary material for: Saproxylic beetles’ morphological traits and higher trophic guilds indicate boreal forest naturalness

Ross Wetherbee^1,2^*, Tone Birkemoe^2^, Ryan C. Burner^3,2^, Anne Sverdrup-Thygeson^2^

^1^Department of Environmental Sciences, Western Norway University of Applied Sciences, Sogndal, Norway

^2^Faculty of Environmental Sciences and Natural Resource Management, Norwegian University of Life Sciences, Ås Norway

^3^U.S. Geological Survey, Upper Midwest Environmental Sciences Center, La Crosse, WI, USA

*Corresponding author

E-mail: [ross.wetherbee@hvl.no](mailto:ross.wetherbee@hvl.no)

Postal address: Røyrgata 6, 6856 Sogndal, Norway

**Keywords:** Saproxylic beetles, biodiversity monitoring, forest management, functional traits, community composition, species richness

**Acknowledgements**

We would like to thank Sindre Ligaard for identifying the beetle species, and the numerous field assistants that contributed to trapping efforts. We also extend our appreciation to the two anonymous reviewers and Dr. Molly Van Appledorn for their valuable comments on the manuscript. Any use of trade, firm, or product names is for descriptive purposes only and does not imply endorsement by the U.S. Government.

**SUPPLEMENTAL MATERIAL**

**Figures**


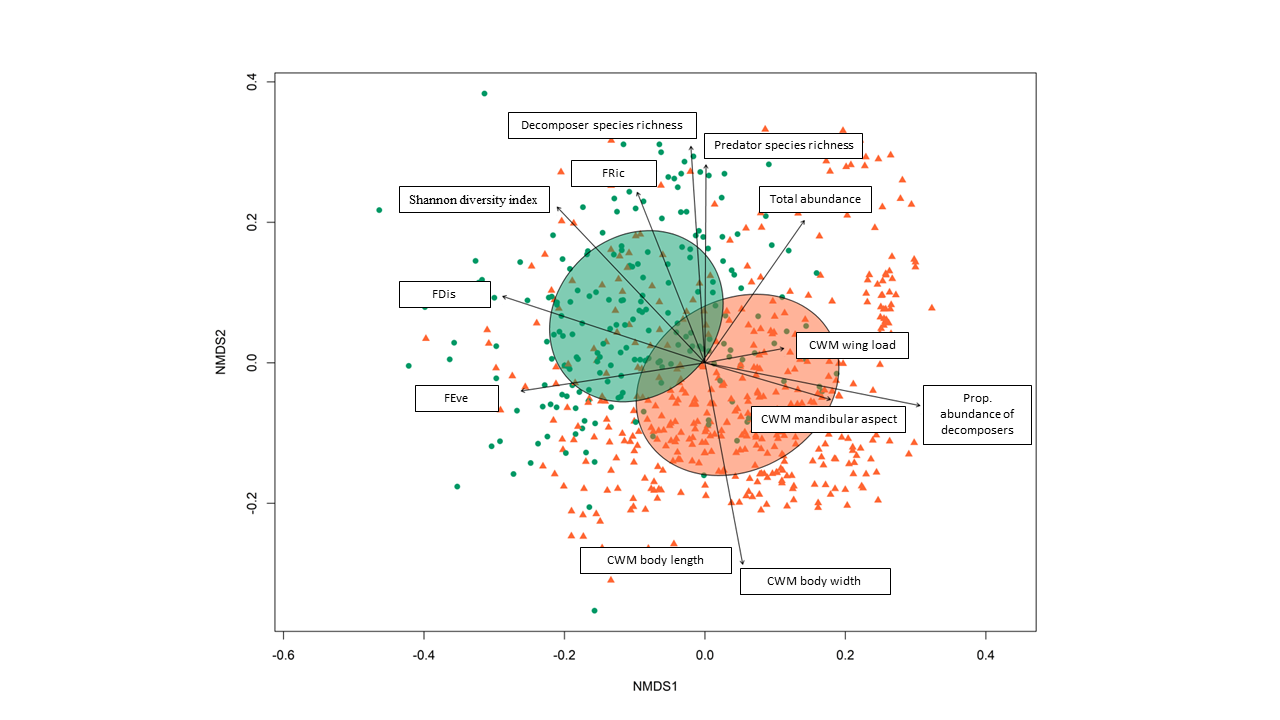


**Figure S1:** A plot of the first two dimensions of the non-metric multidimensional scaling (NMDS) ordination of all study sites. Saproxylic beetles were sampled with flight intercept traps in either near-natural (green circles) or managed forests (orange triangles). The 95% confidence ellipse for each forest type is shown, and the additional measures of beetle biodiversity are plotted as vectors. All variables were related to ordination axes more than by random chance (alpha < 0.05, see Table 2 for model results).

**
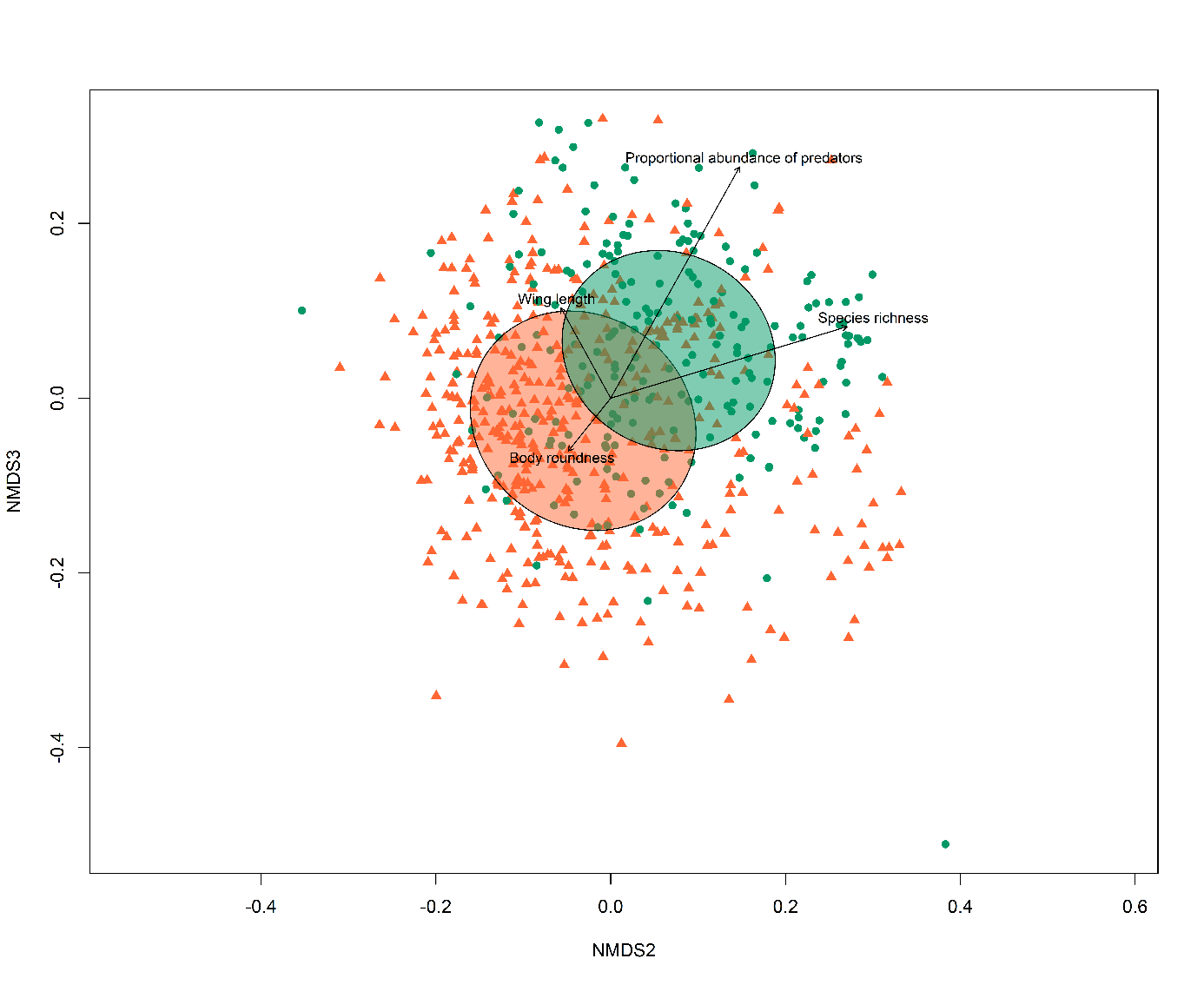
Figure S2:** A plot of the 2^nd^ and 3^rd^ dimensions of the non-metric multidimensional scaling (NMDS) ordination of all study sites. Saproxylic beetles were sampled with flight intercept traps in either near-natural (green circles) or managed forests (orange triangles). The 95% confidence ellipse for each forest type is shown, and the main differences in beetle biodiversity are plotted as vectors.

**

**

**Figure S3:** Shepard plot for nonmetric multidimensional scaling (NMDS) results. The plot shows the ordination distances and monotone line against original dissimilarities. The ‘nonmetric fit’ (top left text) is based on stress (defined as R2 = 1-S*S) and the ‘linear fit’ is the squared correlation between fitted values and ordination distances.

**Tables**

**Table S1:** Trapping locations in southeastern Norway with site identification numbers, coordinates, trap tree type, forest management category, and year(s) sampled. Traps were either attached to the trunk of a focal tree, Eurasian aspen (Aspen) or Norway spruce (Spruce), or were free hanging (None) in the forests. Managed forests are mature forests managed with stand-replacing clear-cutting, whereas near-natural forests are previously managed forests that have never been clear-cut and are now developing natural forest characteristics.

| Site | Dataset | XUTM32N | YUTM32N | Trap tree | Forest management  category | Years |
| --- | --- | --- | --- | --- | --- | --- |
| s199800001 | Dr.Scient | 611656 | 6637212 | Aspen | Managed | 1998 |
| s199800002 | Dr.Scient | 611253 | 6637250 | Aspen | Managed | 1998 |
| s199800003 | Dr.Scient | 611399 | 6637217 | Aspen | Managed | 1998 |
| s199800004 | Dr.Scient | 611333 | 6637114 | Aspen | Managed | 1998 |
| s199800005 | Dr.Scient | 611806 | 6635577 | Aspen | Managed | 1998 |
| s199800006 | Dr.Scient | 611386 | 6635364 | Aspen | Managed | 1998 |
| s199800007 | Dr.Scient | 611245 | 6635474 | Aspen | Managed | 1998 |
| s199800008 | Dr.Scient | 611860 | 6635113 | Aspen | Managed | 1998 |
| s199800009 | Dr.Scient | 612130 | 6635113 | Aspen | Managed | 1998 |
| s199800010 | Dr.Scient | 612364 | 6634925 | Aspen | Managed | 1998 |
| s199800012 | Dr.Scient | 611997 | 6642092 | Aspen | Managed | 1998 |
| s199800013 | Dr.Scient | 611993 | 6642593 | Aspen | Managed | 1998 |
| s199800014 | Dr.Scient | 611884 | 6642436 | Aspen | Managed | 1998 |
| s199800015 | Dr.Scient | 611647 | 6637956 | Aspen | Managed | 1998 |
| s199800016 | Dr.Scient | 611327 | 6636934 | Aspen | Managed | 1998 |
| s199800017 | Dr.Scient | 612516 | 6635508 | Aspen | Managed | 1998 |
| s199800019 | Dr.Scient | 611837 | 6637421 | Aspen | Managed | 1998 |
| s199800020 | Dr.Scient | 611741 | 6640309 | Aspen | Managed | 1998 |
| s199800021 | Dr.Scient | 612307 | 6639843 | Aspen | Managed | 1998 |
| s199800022 | Dr.Scient | 612432 | 6639740 | Aspen | Managed | 1998 |
| s199800023 | Dr.Scient | 613255 | 6640813 | Aspen | Managed | 1998 |
| s199800024 | Dr.Scient | 609617 | 6636621 | Aspen | Managed | 1998 |
| s199800025 | Dr.Scient | 609722 | 6636928 | Aspen | Managed | 1998 |
| s199800026 | Dr.Scient | 609519 | 6636461 | Aspen | Managed | 1998 |
| s199800027 | Dr.Scient | 609314 | 6637007 | Aspen | Managed | 1998 |
| s199800028 | Dr.Scient | 610296 | 6639984 | Aspen | Managed | 1998 |
| s199800029 | Dr.Scient | 612732 | 6638609 | Aspen | Managed | 1999 |
| s199800030 | Dr.Scient | 612755 | 6638686 | Aspen | Managed | 1999 |
| s199800031 | Dr.Scient | 609147 | 6637179 | Aspen | Managed | 1999 |
| s199800032 | Dr.Scient | 610368 | 6635511 | Aspen | Managed | 1999 |
| s199800033 | Dr.Scient | 610202 | 6635675 | Aspen | Managed | 1999 |
| s199800039 | Dr.Scient | 609368 | 6638258 | Aspen | Managed | 1999 |
| s199800040 | Dr.Scient | 609318 | 6638296 | Aspen | Managed | 1999 |
| s200400100 | Gran.livsl | 629259 | 6783465 | Spruce | Managed | 2005 |
| s200400101 | Gran.livsl | 629235 | 6783498 | Spruce | Managed | 2005 |
| s200400102 | Gran.livsl | 629245 | 6783528 | Spruce | Managed | 2005 |
| s200400103 | Gran.livsl | 629249 | 6783575 | Spruce | Managed | 2005 |
| s200400104 | Gran.livsl | 629245 | 6783595 | Spruce | Managed | 2005 |
| s200400105 | Gran.livsl | 629254 | 6783653 | Spruce | Managed | 2005 |
| s200400106 | Gran.livsl | 629219 | 6783715 | Spruce | Managed | 2005 |
| s200400107 | Gran.livsl | 629141 | 6783743 | Spruce | Managed | 2005 |
| s200400108 | Gran.livsl | 629097 | 6783705 | Spruce | Managed | 2005 |
| s200400109 | Gran.livsl | 629108 | 6783705 | Spruce | Managed | 2005 |
| s200400110 | Gran.livsl | 629090 | 6783762 | Spruce | Managed | 2005 |
| s200400111 | Gran.livsl | 629164 | 6783812 | Spruce | Managed | 2005 |
| s200400112 | Gran.livsl | 629260 | 6783816 | Spruce | Managed | 2005 |
| s200400113 | Gran.livsl | 629280 | 6783842 | Spruce | Managed | 2005 |
| s200400114 | Gran.livsl | 629467 | 6783891 | Spruce | Managed | 2005 |
| s200400115 | Gran.livsl | 629490 | 6783878 | Spruce | Managed | 2005 |
| s200400116 | Gran.livsl | 629483 | 6783969 | Spruce | Managed | 2005 |
| s200400117 | Gran.livsl | 629483 | 6783985 | Spruce | Managed | 2005 |
| s200400118 | Gran.livsl | 629290 | 6784066 | Spruce | Managed | 2005 |
| s200400119 | Gran.livsl | 629309 | 6784042 | Spruce | Managed | 2005 |
| s200400120 | Gran.livsl | 629356 | 6784038 | Spruce | Managed | 2005 |
| s200400121 | Gran.livsl | 629382 | 6784044 | Spruce | Managed | 2005 |
| s200400122 | Gran.livsl | 629396 | 6784012 | Spruce | Managed | 2005 |
| s200400123 | Gran.livsl | 629218 | 6783838 | Spruce | Managed | 2005 |
| s200400124 | Gran.livsl | 629210 | 6783855 | Spruce | Managed | 2005 |
| s200400125 | Gran.livsl | 629233 | 6784117 | Spruce | Managed | 2005 |
| s200400126 | Gran.livsl | 629232 | 6784048 | Spruce | Managed | 2005 |
| s200400127 | Gran.livsl | 629216 | 6783927 | Spruce | Managed | 2005 |
| s200400128 | Gran.livsl | 629194 | 6783905 | Spruce | Managed | 2005 |
| s200400129 | Gran.livsl | 629144 | 6783872 | Spruce | Managed | 2005 |
| sK10 | Hurdal | 603604 | 6711380 | Spruce | Managed | 2015 |
| sK11 | Hurdal | 601061 | 6702020 | Spruce | Managed | 2015 |
| sK12 | Hurdal | 606128 | 6699398 | Spruce | Managed | 2015 |
| sK13 | Hurdal | 606956 | 6693811 | Spruce | Managed | 2015 |
| sK15 | Hurdal | 606211 | 6707515 | Spruce | Managed | 2015 |
| sK16 | Hurdal | 600469 | 6705931 | Spruce | Managed | 2015 |
| sK17 | Hurdal | 603631 | 6690051 | Spruce | Managed | 2015 |
| sK18 | Hurdal | 604027 | 6689575 | Spruce | Managed | 2015 |
| sK21 | Hurdal | 606730 | 6692563 | Spruce | Managed | 2015 |
| sK23 | Hurdal | 603337 | 6690591 | Spruce | Managed | 2015 |
| sK25 | Hurdal | 603422 | 6690644 | Spruce | Managed | 2015 |
| sK3 | Hurdal | 606582 | 6687160 | Spruce | Managed | 2015 |
| sK4 | Hurdal | 606488 | 6687183 | Spruce | Managed | 2015 |
| sK9 | Hurdal | 607286 | 6702488 | Spruce | Managed | 2015 |
| s200700121 | Hverdagshensyn&vernebehov | 610209 | 6638445 | Aspen | Managed | 2007, ‘08, ‘09 |
| s200700122 | Hverdagshensyn&vernebehov | 610697 | 6637030 | Aspen | Managed | 2007, ‘08, ‘09 |
| s200700123 | Hverdagshensyn&vernebehov | 611200 | 6636832 | Aspen | Managed | 2007, ‘08, ‘09 |
| s200700125 | Hverdagshensyn&vernebehov | 611936 | 6637205 | Aspen | Managed | 2007, ‘08, ‘09 |
| s200700126 | Hverdagshensyn&vernebehov | 608297 | 6638724 | Aspen | Managed | 2007, ‘08, ‘09 |
| s200700127 | Hverdagshensyn&vernebehov | 608516 | 6638609 | Aspen | Managed | 2007, ‘08, ‘09 |
| s200700128 | Hverdagshensyn&vernebehov | 608837 | 6639203 | Aspen | Managed | 2007, ‘08, ‘09 |
| s200700221 | Hverdagshensyn&vernebehov | 594321 | 6653371 | Aspen | Managed | 2007, ‘08, ‘09 |
| s200700222 | Hverdagshensyn&vernebehov | 594232 | 6653201 | Aspen | Managed | 2007, ‘08, ‘09 |
| s200700223 | Hverdagshensyn&vernebehov | 595596 | 6655086 | Aspen | Managed | 2007, ‘08, ‘09 |
| s200700224 | Hverdagshensyn&vernebehov | 595832 | 6656376 | Aspen | Managed | 2007, ‘08, ‘09 |
| s200700225 | Hverdagshensyn&vernebehov | 596146 | 6656629 | Aspen | Managed | 2007, ‘08, ‘09 |
| s200700226 | Hverdagshensyn&vernebehov | 596135 | 6656779 | Aspen | Managed | 2007, ‘08, ‘09 |
| s200700227 | Hverdagshensyn&vernebehov | 595001 | 6658691 | Aspen | Managed | 2007, ‘08, ‘09 |
| s200700228 | Hverdagshensyn&vernebehov | 595164 | 6658689 | Aspen | Managed | 2007, ‘08, ‘09 |
| s200700321 | Hverdagshensyn&vernebehov | 564295 | 6616443 | Aspen | Managed | 2007, ‘08, ‘09 |
| s200700322 | Hverdagshensyn&vernebehov | 561202 | 6613639 | Aspen | Managed | 2007, ‘08, ‘09 |
| s200700323 | Hverdagshensyn&vernebehov | 561681 | 6613811 | Aspen | Managed | 2007, ‘08, ‘09 |
| s200700324 | Hverdagshensyn&vernebehov | 561594 | 6613665 | Aspen | Managed | 2007, ‘08, ‘09 |
| s200700325 | Hverdagshensyn&vernebehov | 561404 | 6612790 | Aspen | Managed | 2007, ‘08, ‘09 |
| s200700327 | Hverdagshensyn&vernebehov | 562419 | 6610885 | Aspen | Managed | 2007, ‘08, ‘09 |
| s200700328 | Hverdagshensyn&vernebehov | 562485 | 6611276 | Aspen | Managed | 2007, ‘08, ‘09 |
| s200100101 | Kostnadseffekt | 611828 | 6636270 | Aspen | Managed | 2001, ‘02, ‘03, ‘04, ‘05, ‘13 |
| s200100102 | Kostnadseffekt | 608776 | 6635034 | Aspen | Managed | 2001, ‘02, ‘03, ‘04, ‘05, ‘13 |
| s200100103 | Kostnadseffekt | 608702 | 6635139 | Aspen | Managed | 2001, ‘02, ‘03, ‘04, ‘05 |
| s200100104 | Kostnadseffekt | 608687 | 6635215 | Aspen | Managed | 2001, ‘02, ‘03, ‘04, ‘05, ‘13 |
| s200100105 | Kostnadseffekt | 608405 | 6636562 | Aspen | Managed | 2001, ‘02, ‘03, ‘04, ‘05, ‘13 |
| s200100106 | Kostnadseffekt | 608442 | 6636634 | Aspen | Managed | 2001, ‘02, ‘03, ‘04, ‘05, ‘13 |
| s200100107 | Kostnadseffekt | 608402 | 6636720 | Aspen | Managed | 2001, ‘02, ‘03, ‘04, ‘05, ‘13 |
| s200100108 | Kostnadseffekt | 612172 | 6635467 | Aspen | Managed | 2001, ‘02, ‘03, ‘04, ‘05, ‘13 |
| s200100109 | Kostnadseffekt | 612095 | 6635468 | Aspen | Managed | 2001, ‘02, ‘03, ‘04, ‘05, ‘13 |
| s200100110 | Kostnadseffekt | 612438 | 6635626 | Aspen | Managed | 2001, ‘02, ‘03, ‘04, ‘05, ‘13 |
| s200100111 | Kostnadseffekt | 612461 | 6635530 | Aspen | Managed | 2001, ‘02, ‘03, ‘04, ‘05, ‘13 |
| s200100112 | Kostnadseffekt | 611902 | 6636201 | Aspen | Managed | 2001, ‘02, ‘03, ‘04, ‘05, ‘13 |
| s200100113 | Kostnadseffekt | 611933 | 6636288 | Aspen | Managed | 2001, ‘02, ‘03, ‘04, ‘05, ‘13 |
| s200100114 | Kostnadseffekt | 611991 | 6636334 | Aspen | Managed | 2001, ‘02, ‘03 |
| s200100115 | Kostnadseffekt | 611894 | 6636365 | Aspen | Managed | 2001, ‘02, ‘03, ‘04, ‘05, ‘13 |
| s200100301 | Kostnadseffekt | 587083 | 6657449 | Aspen | Managed | 2001, ‘02, ‘03, ‘04, ‘05 |
| s200100302 | Kostnadseffekt | 587048 | 6657568 | Aspen | Managed | 2001, ‘02, ‘03, ‘04, ‘05 |
| s200100303 | Kostnadseffekt | 582316 | 6654262 | Aspen | Managed | 2001, ‘02, ‘03, ‘04, ‘05 |
| s200100304 | Kostnadseffekt | 582197 | 6654276 | Aspen | Managed | 2001, ‘02, ‘03, ‘04, ‘05 |
| s200100305 | Kostnadseffekt | 582043 | 6654624 | Aspen | Managed | 2001, ‘02, ‘03, ‘04, ‘05 |
| s200100306 | Kostnadseffekt | 582044 | 6654707 | Aspen | Managed | 2001, ‘02, ‘03, ‘04, ‘05 |
| s200100307 | Kostnadseffekt | 582077 | 6654987 | Aspen | Managed | 2001, ‘02, ‘03, ‘04, ‘05 |
| s200100308 | Kostnadseffekt | 582059 | 6655077 | Aspen | Managed | 2001, ‘02, ‘03, ‘04, ‘05 |
| s200100309 | Kostnadseffekt | 588078 | 6658143 | Aspen | Managed | 2001, ‘02, ‘03, ‘04, ‘05 |
| s200100310 | Kostnadseffekt | 588161 | 6658540 | Aspen | Managed | 2001, ‘02, ‘03, ‘04, ‘05 |
| s200100311 | Kostnadseffekt | 587832 | 6658389 | Aspen | Managed | 2001, ‘02, ‘03, ‘04, ‘05 |
| s200100312 | Kostnadseffekt | 587779 | 6658457 | Aspen | Managed | 2001, ‘02, ‘03, ‘04, ‘05 |
| s200100313 | Kostnadseffekt | 588393 | 6664098 | Aspen | Managed | 2001, ‘02, ‘03, ‘04, ‘05 |
| s200100314 | Kostnadseffekt | 588264 | 6664139 | Aspen | Managed | 2001, ‘02, ‘03, ‘04, ‘05 |
| s200100315 | Kostnadseffekt | 588309 | 6664209 | Aspen | Managed | 2001, ‘02, ‘03, ‘04, ‘05 |
| s200100501 | Kostnadseffekt | 587337 | 6657210 | Aspen | Managed | 2001, ‘02, ‘03, ‘04 |
| s200100502 | Kostnadseffekt | 587322 | 6657191 | Aspen | Managed | 2001, ‘02, ‘03, ‘04 |
| s200100503 | Kostnadseffekt | 587319 | 6657200 | Aspen | Managed | 2001, ‘02, ‘03, ‘04 |
| s200100504 | Kostnadseffekt | 587526 | 6657272 | Aspen | Managed | 2001, ‘02, ‘03, ‘04 |
| s200100505 | Kostnadseffekt | 587349 | 6657726 | Aspen | Managed | 2001, ‘02, ‘03, ‘04 |
| s200100506 | Kostnadseffekt | 587238 | 6657462 | Aspen | Managed | 2001, ‘02, ‘03, ‘04 |
| s200100601 | Kostnadseffekt | 587157 | 6657753 | Aspen | Managed | 2001, ‘02, ‘03, ‘04 |
| s200100602 | Kostnadseffekt | 587158 | 6657746 | Aspen | Managed | 2001, ‘02, ‘03, ‘04 |
| s200100603 | Kostnadseffekt | 587045 | 6657587 | Aspen | Managed | 2001, ‘02, ‘03, ‘04 |
| s200100604 | Kostnadseffekt | 587078 | 6657486 | Aspen | Managed | 2001, ‘02, ‘03, ‘04 |
| s200100605 | Kostnadseffekt | 587093 | 6657482 | Aspen | Managed | 2001, ‘02, ‘03, ‘04 |
| s200100606 | Kostnadseffekt | 587103 | 6657450 | Aspen | Managed | 2001, ‘02, ‘03, ‘04 |
| s200100701 | Kostnadseffekt | 612923 | 6639747 | Spruce | Managed | 2005 |
| s200100702 | Kostnadseffekt | 613099 | 6639654 | Spruce | Managed | 2005 |
| s200100703 | Kostnadseffekt | 612220 | 6635506 | Spruce | Managed | 2005 |
| s200100704 | Kostnadseffekt | 612145 | 6635438 | Spruce | Managed | 2005 |
| s200100705 | Kostnadseffekt | 608810 | 6635046 | Spruce | Managed | 2005 |
| s200100706 | Kostnadseffekt | 608845 | 6634976 | Spruce | Managed | 2005 |
| s200100707 | Kostnadseffekt | 608775 | 6635052 | Spruce | Managed | 2005 |
| s200100708 | Kostnadseffekt | 608679 | 6635189 | Spruce | Managed | 2005 |
| s200100709 | Kostnadseffekt | 608688 | 6635263 | Spruce | Managed | 2005 |
| s200100710 | Kostnadseffekt | 608675 | 6635322 | Spruce | Managed | 2005 |
| s200100711 | Kostnadseffekt | 608353 | 6636680 | Spruce | Managed | 2005 |
| s200100712 | Kostnadseffekt | 608452 | 6636768 | Spruce | Managed | 2005 |
| s200100713 | Kostnadseffekt | 608317 | 6636576 | Spruce | Managed | 2005 |
| s200100714 | Kostnadseffekt | 608375 | 6636762 | Spruce | Managed | 2005 |
| s200100715 | Kostnadseffekt | 608364 | 6636823 | Spruce | Managed | 2005 |
| s200101101 | Kostnadseffekt | 611797 | 6636262 | None | Managed | 2001, ‘02, ‘03 |
| s200101102 | Kostnadseffekt | 608788 | 6635051 | None | Managed | 2001, ‘02, ‘03 |
| s200101103 | Kostnadseffekt | 608678 | 6635146 | None | Managed | 2001, ‘02, ‘03 |
| s200101104 | Kostnadseffekt | 608689 | 6635243 | None | Managed | 2001, ‘02, ‘03 |
| s200101105 | Kostnadseffekt | 608398 | 6636523 | None | Managed | 2001, ‘02, ‘03 |
| s200101106 | Kostnadseffekt | 608461 | 6636662 | None | Managed | 2001, ‘02, ‘03 |
| s200101107 | Kostnadseffekt | 608395 | 6636729 | None | Managed | 2001, ‘02, ‘03 |
| s200101108 | Kostnadseffekt | 612147 | 6635447 | None | Managed | 2001, ‘02, ‘03 |
| s200101109 | Kostnadseffekt | 612105 | 6635440 | None | Managed | 2001, ‘02, ‘03 |
| s200101110 | Kostnadseffekt | 612468 | 6635615 | None | Managed | 2001, ‘02, ‘03 |
| s200101111 | Kostnadseffekt | 612464 | 6635494 | None | Managed | 2001, ‘02, ‘03 |
| s200101112 | Kostnadseffekt | 611906 | 6636174 | None | Managed | 2001, ‘02, ‘03 |
| s200101113 | Kostnadseffekt | 611945 | 6636257 | None | Managed | 2001, ‘02, ‘03 |
| s200101114 | Kostnadseffekt | 612012 | 6636356 | None | Managed | 2001, ‘02, ‘03 |
| s200101115 | Kostnadseffekt | 611869 | 6636392 | None | Managed | 2001, ‘02, ‘03 |
| s200400SO7 | ARKO | 501079 | 6549181 | Aspen | Near-natural | 2004 |
| s200400SO8 | ARKO | 501098 | 6549226 | Aspen | Near-natural | 2004 |
| s200400SO9 | ARKO | 501119 | 6549224 | Aspen | Near-natural | 2004 |
| s200400SV5 | ARKO | 500729 | 6548530 | Aspen | Near-natural | 2004 |
| s200400SV6 | ARKO | 500742 | 6548534 | Aspen | Near-natural | 2004 |
| s200400SV7 | ARKO | 500755 | 6548533 | Aspen | Near-natural | 2004 |
| sARK | Billebestem_gammelskog | 641843 | 6719678 | Spruce | Near-natural | 2018, ‘19 |
| sFOL | Billebestem_gammelskog | 509542 | 6616106 | Spruce | Near-natural | 2018, ‘19 |
| sGAU | Billebestem_gammelskog | 611413 | 6622717 | Spruce | Near-natural | 2018, ‘19 |
| sGUL | Billebestem_gammelskog | 600037 | 6692425 | Spruce | Near-natural | 2018, ‘19 |
| sHAL | Billebestem_gammelskog | 541266 | 6655996 | Spruce | Near-natural | 2018, ‘19 |
| sHES | Billebestem_gammelskog | 531042 | 6686104 | Spruce | Near-natural | 2018, ‘19 |
| sKJE | Billebestem_gammelskog | 512114 | 6704077 | Spruce | Near-natural | 2018, ‘19 |
| sNOR | Billebestem_gammelskog | 599094 | 6625236 | Spruce | Near-natural | 2018, ‘19 |
| sOPP | Billebestem_gammelskog | 584049 | 6661890 | Spruce | Near-natural | 2018, ‘19 |
| sOST | Billebestem_gammelskog | 613451 | 6637062 | Spruce | Near-natural | 2018, ‘19 |
| sRAM | Billebestem_gammelskog | 613603 | 6642478 | Spruce | Near-natural | 2018, ‘19 |
| sSKO | Billebestem_gammelskog | 599284 | 6679482 | Spruce | Near-natural | 2018, ‘19 |
| sSON | Billebestem_gammelskog | 589339 | 6617956 | Spruce | Near-natural | 2018 |
| sSPF | Billebestem_gammelskog | 583935 | 6670047 | Spruce | Near-natural | 2018, ‘19 |
| sSPG | Billebestem_gammelskog | 585602 | 6673349 | Spruce | Near-natural | 2018, ‘19 |
| sSTY | Billebestem_gammelskog | 600547 | 6683271 | Spruce | Near-natural | 2018, ‘19 |
| sTOR | Billebestem_gammelskog | 606353 | 6716302 | Spruce | Near-natural | 2018, ‘19 |
| sTRH | Billebestem_gammelskog | 523772 | 6655281 | Spruce | Near-natural | 2018, ‘19 |
| sTRN | Billebestem_gammelskog | 516645 | 6661407 | Spruce | Near-natural | 2018, ‘19 |
| sVIN | Billebestem_gammelskog | 599476 | 6623484 | Spruce | Near-natural | 2018, ‘19 |
| s199800011 | Dr.Scient | 612513 | 6635067 | Aspen | Near-natural | 1998 |
| s199800018 | Dr.Scient | 612798 | 6635479 | Aspen | Near-natural | 1998 |
| s199800034 | Dr.Scient | 612815 | 6635873 | Aspen | Near-natural | 1999 |
| s199800035 | Dr.Scient | 612699 | 6635776 | Aspen | Near-natural | 1999 |
| s199800036 | Dr.Scient | 612752 | 6635737 | Aspen | Near-natural | 1999 |
| s199800037 | Dr.Scient | 612914 | 6635441 | Aspen | Near-natural | 1999 |
| s199800038 | Dr.Scient | 612938 | 6635523 | Aspen | Near-natural | 1999 |
| sN1 | Hurdal | 607658 | 6684775 | Spruce | Near-natural | 2015 |
| sN11 | Hurdal | 606720 | 6685506 | Spruce | Near-natural | 2015 |
| sN12 | Hurdal | 602717 | 6690452 | Spruce | Near-natural | 2015 |
| sN13 | Hurdal | 600049 | 6705719 | Spruce | Near-natural | 2015 |
| sN16 | Hurdal | 600507 | 6705820 | Spruce | Near-natural | 2015 |
| sN19 | Hurdal | 606829 | 6693923 | Spruce | Near-natural | 2015 |
| sN2 | Hurdal | 606227 | 6688084 | Spruce | Near-natural | 2015 |
| sN20 | Hurdal | 604030 | 6690285 | Spruce | Near-natural | 2015 |
| sN21 | Hurdal | 603763 | 6690225 | Spruce | Near-natural | 2015 |
| sN3 | Hurdal | 606621 | 6687766 | Spruce | Near-natural | 2015 |
| sN4 | Hurdal | 606368 | 6688157 | Spruce | Near-natural | 2015 |
| sN5 | Hurdal | 606719 | 6685973 | Spruce | Near-natural | 2015 |
| sN6 | Hurdal | 604837 | 6685706 | Spruce | Near-natural | 2015 |
| sN8 | Hurdal | 601742 | 6694029 | Spruce | Near-natural | 2015 |
| sN9 | Hurdal | 607424 | 6688120 | Spruce | Near-natural | 2015 |
| s200700111 | Hverdagshensyn&vernebehov | 612207 | 6637287 | Aspen | Near-natural | 2007, ‘08, ‘09 |
| s200700112 | Hverdagshensyn&vernebehov | 610305 | 6636838 | Aspen | Near-natural | 2007, ‘08, ‘09 |
| s200700113 | Hverdagshensyn&vernebehov | 609570 | 6633495 | Aspen | Near-natural | 2007, ‘08, ‘09 |
| s200700114 | Hverdagshensyn&vernebehov | 612187 | 6633242 | Aspen | Near-natural | 2007, ‘08, ‘09 |
| s200700115 | Hverdagshensyn&vernebehov | 610557 | 6633117 | Aspen | Near-natural | 2007, ‘08, ‘09 |
| s200700116 | Hverdagshensyn&vernebehov | 608367 | 6636644 | Aspen | Near-natural | 2007, ‘08, ‘09 |
| s200700117 | Hverdagshensyn&vernebehov | 613084 | 6638955 | Aspen | Near-natural | 2007, ‘08, ‘09 |
| s200700118 | Hverdagshensyn&vernebehov | 611108 | 6638659 | Aspen | Near-natural | 2007, ‘08, ‘09 |
| s200700131 | Hverdagshensyn&vernebehov | 613205 | 6635862 | Aspen | Near-natural | 2007, ‘08, ‘09 |
| s200700132 | Hverdagshensyn&vernebehov | 613242 | 6635550 | Aspen | Near-natural | 2007, ‘08, ‘09 |
| s200700133 | Hverdagshensyn&vernebehov | 614399 | 6635858 | Aspen | Near-natural | 2007, ‘08, ‘09 |
| s200700134 | Hverdagshensyn&vernebehov | 614086 | 6636455 | Aspen | Near-natural | 2007, ‘08, ‘09 |
| s200700135 | Hverdagshensyn&vernebehov | 613524 | 6636779 | Aspen | Near-natural | 2007, ‘08, ‘09 |
| s200700136 | Hverdagshensyn&vernebehov | 612845 | 6636507 | Aspen | Near-natural | 2007, ‘08, ‘09 |
| s200700137 | Hverdagshensyn&vernebehov | 613463 | 6636955 | Aspen | Near-natural | 2007, ‘08, ‘09 |
| s200700138 | Hverdagshensyn&vernebehov | 613542 | 6637026 | Aspen | Near-natural | 2007, ‘08, ‘09 |
| s200700211 | Hverdagshensyn&vernebehov | 593370 | 6656758 | Aspen | Near-natural | 2007, ‘08, ‘09 |
| s200700212 | Hverdagshensyn&vernebehov | 597035 | 6652266 | Aspen | Near-natural | 2007, ‘08, ‘09 |
| s200700213 | Hverdagshensyn&vernebehov | 594236 | 6659334 | Aspen | Near-natural | 2007, ‘08, ‘09 |
| s200700214 | Hverdagshensyn&vernebehov | 593938 | 6657074 | Aspen | Near-natural | 2007, ‘08, ‘09 |
| s200700215 | Hverdagshensyn&vernebehov | 594415 | 6653600 | Aspen | Near-natural | 2007, ‘08, ‘09 |
| s200700216 | Hverdagshensyn&vernebehov | 597251 | 6659034 | Aspen | Near-natural | 2007, ‘08, ‘09 |
| s200700217 | Hverdagshensyn&vernebehov | 594113 | 6653023 | Aspen | Near-natural | 2007, ‘08, ‘09 |
| s200700218 | Hverdagshensyn&vernebehov | 595158 | 6652427 | Aspen | Near-natural | 2007, ‘08, ‘09 |
| s200700231 | Hverdagshensyn&vernebehov | 597882 | 6658074 | Aspen | Near-natural | 2007, ‘08, ‘09 |
| s200700232 | Hverdagshensyn&vernebehov | 599693 | 6658091 | Aspen | Near-natural | 2007, ‘08, ‘09 |
| s200700233 | Hverdagshensyn&vernebehov | 597569 | 6658096 | Aspen | Near-natural | 2007, ‘08, ‘09 |
| s200700234 | Hverdagshensyn&vernebehov | 599342 | 6658074 | Aspen | Near-natural | 2007, ‘08, ‘09 |
| s200700235 | Hverdagshensyn&vernebehov | 599386 | 6657174 | Aspen | Near-natural | 2007, ‘08, ‘09 |
| s200700236 | Hverdagshensyn&vernebehov | 599358 | 6658416 | Aspen | Near-natural | 2007, ‘08, ‘09 |
| s200700237 | Hverdagshensyn&vernebehov | 599069 | 6657196 | Aspen | Near-natural | 2007, ‘08, ‘09 |
| s200700238 | Hverdagshensyn&vernebehov | 598169 | 6657496 | Aspen | Near-natural | 2007, ‘08, ‘09 |
| s200700311 | Hverdagshensyn&vernebehov | 561543 | 6612214 | Aspen | Near-natural | 2007, ‘08, ‘09 |
| s200700312 | Hverdagshensyn&vernebehov | 562003 | 6612006 | Aspen | Near-natural | 2007, ‘08, ‘09 |
| s200700313 | Hverdagshensyn&vernebehov | 559452 | 6612871 | Aspen | Near-natural | 2007, ‘08, ‘09 |
| s200700314 | Hverdagshensyn&vernebehov | 563958 | 6615743 | Aspen | Near-natural | 2007, ‘08, ‘09 |
| s200700315 | Hverdagshensyn&vernebehov | 561591 | 6617628 | Aspen | Near-natural | 2007, ‘08, ‘09 |
| s200700316 | Hverdagshensyn&vernebehov | 561144 | 6614257 | Aspen | Near-natural | 2007, ‘08, ‘09 |
| s200700317 | Hverdagshensyn&vernebehov | 563170 | 6614132 | Aspen | Near-natural | 2007, ‘08 |
| s200700318 | Hverdagshensyn&vernebehov | 561492 | 6615513 | Aspen | Near-natural | 2007, ‘08, ‘09 |
| s200700326 | Hverdagshensyn&vernebehov | 599358 | 6658416 | Aspen | Near-natural | 2007, ‘08, ‘09 |
| s200700331 | Hverdagshensyn&vernebehov | 559977 | 6611418 | Aspen | Near-natural | 2007, ‘08, ‘09 |
| s200700332 | Hverdagshensyn&vernebehov | 559677 | 6611136 | Aspen | Near-natural | 2007, ‘08, ‘09 |
| s200700333 | Hverdagshensyn&vernebehov | 559977 | 6611136 | Aspen | Near-natural | 2007, ‘08, ‘09 |
| s200700334 | Hverdagshensyn&vernebehov | 560277 | 6612036 | Aspen | Near-natural | 2007, ‘08, ‘09 |
| s200700335 | Hverdagshensyn&vernebehov | 559576 | 6611343 | Aspen | Near-natural | 2007, ‘08, ‘09 |
| s200700336 | Hverdagshensyn&vernebehov | 559654 | 6610940 | Aspen | Near-natural | 2007, ‘08, ‘09 |
| s200700337 | Hverdagshensyn&vernebehov | 560243 | 6610866 | Aspen | Near-natural | 2007, ‘08, ‘09 |
| s200700338 | Hverdagshensyn&vernebehov | 559265 | 6611329 | Aspen | Near-natural | 2007, ‘08, ‘09 |
| sMJT | PathExt | 491501 | 6571992 | Spruce | Near-natural | 2014 |
| sMJWT | PathExt | 507465 | 6564645 | Spruce | Near-natural | 2014 |

**Table S2:** Species list

| **Species name** |
| --- |
| \| Abdera flexuosa \| \| --- \| \| Acmaeops septentrionis \| \| Agaricochara latissima \| \| Agathidium nigripenne \| \| Alosterna tabacicolor \| \| Ampedus balteatus \| \| Ampedus cinnabarinus \| \| Ampedus nigrinus \| \| Ampedus nigroflavus \| \| Ampedus pomorum \| \| Ampedus tristis \| \| Anaspis bohemica \| \| Anaspis flava \| \| Anaspis frontalis \| \| Anaspis marginicollis \| \| Anaspis rufilabris \| \| Anaspis thoracica \| \| Anastrangalia reyi \| \| Anastrangalia sanguinolenta \| \| Anisandrus dispar \| \| Anisotoma axillaris \| \| Anisotoma castanea \| \| Anisotoma glabra \| \| Anisotoma humeralis \| \| Anisotoma orbicularis \| \| Anomognathus cuspidatus \| \| Anthaxia morio \| \| Anthaxia quadripunctata \| \| Anthribus nebulosus \| \| Aplocnemus nigricornis \| \| Aplocnemus tarsalis \| \| Asemum striatum \| \| Athous subfuscus \| \| Atomaria affinis \| \| Atomaria alpina \| \| Atomaria badia \| \| Atomaria bella \| \| Atomaria diluta \| \| Atomaria subangulata \| \| Atomaria umbrina \| \| Atrecus affinis \| \| Atrecus longiceps \| \| Atrecus pilicornis \| \| Bibloporus bicolor \| \| Bibloporus minutus \| \| Bisnius subuliformis \| \| Bitoma crenata \| \| Bolitochara mulsanti \| \| Bolitophagus reticulatus \| \| Buprestis rustica \| \| Cacotemnus thomsoni \| \| Callidium coriaceum \| \| Cardiophorus ruficollis \| \| Cerylon deplanatum \| \| Cerylon fagi \| \| Cerylon ferrugineum \| \| Cerylon histeroides \| \| Chrysanthia geniculata \| \| Cis bidentatus \| \| Cis boleti \| \| Cis castaneus \| \| Cis comptus \| \| Cis dentatus \| \| Cis fagi \| \| Cis festivus \| \| Cis glabratus \| \| Cis jacquemartii \| \| Cis lineatocribratus \| \| Cis micans \| \| Cis punctulatus \| \| Cis quadridens \| \| Cis vestitus \| \| Cis villosulus \| \| Corticaria lapponica \| \| Corticaria lateritia \| \| Corticaria orbicollis \| \| Corticaria polypori \| \| Corticeus linearis \| \| Corticeus suturalis \| \| Cryphalus asperatus \| \| Cryptolestes abietis \| \| Cryptophagus badius \| \| Cryptophagus fuscicornis \| \| Cryptophagus micaceus \| \| Crypturgus cinereus \| \| Crypturgus hispidulus \| \| Crypturgus pusillus \| \| Ctesias serra \| \| Cucujus cinnaberinus \| \| Curtimorda maculosa \| \| Cybocephalus politus \| \| Cyphea curtula \| \| Dacne bipustulata \| \| Dadobia immersa \| \| Dasytes caeruleus \| \| Dasytes niger \| \| Dasytes obscurus \| \| Dasytes plumbeus \| \| Dendroctonus micans \| \| Dendrophagus crenatus \| \| Denticollis linearis \| \| Diacanthous undulatus \| \| Dictyoptera aurora \| \| Dinaraea aequata \| \| Dinaraea arcana \| \| Dinaraea linearis \| \| Dolichocis laricinus \| \| Dorcatoma dresdensis \| \| Dorcatoma punctulata \| \| Dorcatoma robusta \| \| Dryocoetes alni \| \| Dryocoetes autographus \| \| Dryocoetes villosus \| \| Dryophilus pusillus \| \| Dryophthorus corticalis \| \| Endomychus coccineus \| \| Enicmus fungicola \| \| Enicmus planipennis \| \| Enicmus rugosus \| \| Enicmus testaceus \| \| Ennearthron cornutum \| \| Epuraea angustula \| \| Epuraea boreella \| \| Epuraea deubeli \| \| Epuraea laeviuscula \| \| Epuraea marseuli \| \| Epuraea muehli \| \| Epuraea neglecta \| \| Epuraea oblonga \| \| Epuraea pallescens \| \| Epuraea pygmaea \| \| Epuraea silacea \| \| Epuraea thoracica \| \| Ernobius angusticollis \| \| Ernobius explanatus \| \| Ernobius mollis \| \| Ernobius nigrinus \| \| Ernoporus tiliae \| \| Euglenes pygmaeus \| \| Euplectus bescidicus \| \| Euplectus brunneus \| \| Euplectus decipiens \| \| Euplectus mutator \| \| Euplectus punctatus \| \| Euryusa castanoptera \| \| Evodinus borealis \| \| Gaurotes virginea \| \| Glischrochilus hortensis \| \| Glischrochilus quadripunctatus \| \| Gyrophaena boleti \| \| Gyrophaena manca \| \| Gyrophaena strictula \| \| Hadrobregmus pertinax \| \| Hallomenus axillaris \| \| Hallomenus binotatus \| \| Hapalaraea pygmaea \| \| Hemicoelus canaliculatus \| \| Homalota plana \| \| Hylastes brunneus \| \| Hylastes cunicularius \| \| Hylastes opacus \| \| Hylis cariniceps \| \| Hylis procerulus \| \| Hylobius abietis \| \| Hylobius piceus \| \| Hylobius pinastri \| \| Hylurgops glabratus \| \| Hylurgops palliatus \| \| Ipidia binotata \| \| Ips acuminatus \| \| Ips typographus \| \| Judolia sexmaculata \| \| Latridius hirtus \| \| Leiestes seminiger \| \| Leptophloeus alternans \| \| Leptura quadrifasciata \| \| Leptusa fumida \| \| Leptusa pulchella \| \| Lordithon speciosus \| \| Lordithon trimaculatus \| \| Lygistopterus sanguineus \| \| Magdalis carbonaria \| \| Magdalis duplicata \| \| Magdalis ruficornis \| \| Malthinus biguttatus \| \| Malthinus flaveolus \| \| Malthodes crassicornis \| \| Malthodes flavoguttatus \| \| Malthodes fuscus \| \| Malthodes guttifer \| \| Malthodes marginatus \| \| Malthodes mysticus \| \| Malthodes spathifer \| \| Melanotus castanipes \| \| Microrhagus lepidus \| \| Microscydmus minimus \| \| Microscydmus nanus \| \| Molorchus minor \| \| Mordella holomelaena \| \| Mordellochroa abdominalis \| \| Mycetina cruciata \| \| Mycetochara axillaris \| \| Mycetochara flavipes \| \| Mycetochara obscura \| \| Mycetophagus fulvicollis \| \| Mycetophagus piceus \| \| Mycetophagus populi \| \| Necydalis major \| \| Nemadus colonoides \| \| Nemozoma elongatum \| \| Nudobius lentus \| \| Octotemnus glabriculus \| \| Orchesia micans \| \| Orchesia minor \| \| Orchesia undulata \| \| Orthocis alni \| \| Orthotomicus proximus \| \| Orthotomicus suturalis \| \| Oxymirus cursor \| \| Oxypoda arborea \| \| Pachyta lamed \| \| Paromalus parallelepipedus \| \| Peltis ferruginea \| \| Pentanota meuseli \| \| Phloeocharis subtilissima \| \| Phloeonomus punctipennis \| \| Phloeonomus pusillus \| \| Phloeophagus turbatus \| \| Phloeopora corticalis \| \| Phloeopora testacea \| \| Phloeostiba lapponica \| \| Phloeostiba plana \| \| Phloeotribus spinulosus \| \| Phyllodrepa melanocephala \| \| Phymatura brevicollis \| \| Pissodes castaneus \| \| Pissodes gyllenhalii \| \| Pissodes harcyniae \| \| Pissodes pini \| \| Pityogenes bidentatus \| \| Pityogenes chalcographus \| \| Pityogenes quadridens \| \| Pityophagus ferrugineus \| \| Pityophthorus micrographus \| \| Placusa atrata \| \| Placusa depressa \| \| Placusa incompleta \| \| Placusa suecica \| \| Placusa tachyporoides \| \| Platycerus caraboides \| \| Platycis minutus \| \| Platysoma deplanatum \| \| Platysoma lineare \| \| Platystomos albinus \| \| Plegaderus caesus \| \| Plegaderus vulneratus \| \| Podistra schoenherri \| \| Poecilonota variolosa \| \| Pogonocherus fasciculatus \| \| Polygraphus poligraphus \| \| Polygraphus subopacus \| \| Pseudocistela ceramboides \| \| Ptenidium turgidum \| \| Pteryngium crenatum \| \| Pteryx splendens \| \| Pteryx suturalis \| \| Ptinus dubius \| \| Ptinus subpillosus \| \| Pyropterus nigroruber \| \| Pytho depressus \| \| Quedius brevicornis \| \| Quedius microps \| \| Rabocerus foveolatus \| \| Rhagium inquisitor \| \| Rhagium mordax \| \| Rhizophagus cribratus \| \| Rhizophagus depressus \| \| Rhizophagus dispar \| \| Rhizophagus fenestralis \| \| Rhizophagus ferrugineus \| \| Rhizophagus grandis \| \| Rhizophagus nitidulus \| \| Rhizophagus picipes \| \| Rhyncolus ater \| \| Rhyncolus elongatus \| \| Rhyncolus sculpturatus \| \| Salpingus planirostris \| \| Salpingus ruficollis \| \| Saperda perforata \| \| Scaphisoma assimile \| \| Scaphisoma boleti \| \| Scaphisoma boreale \| \| Scaphisoma subalpinum \| \| Schizotus pectinicornis \| \| Scolytus rugulosus \| \| Scydmoraphes minutus \| \| Sericus brunneus \| \| Serropalpus barbatus \| \| Silvanoprus fagi \| \| Sinodendron cylindricum \| \| Soronia grisea \| \| Soronia punctatissima \| \| Sphindus dubius \| \| Stenichnus bicolor \| \| Stenichnus godarti \| \| Stenurella melanura \| \| Stictoleptura rubra \| \| Strophosoma capitatum \| \| Sulcacis fronticornis \| \| Sulcacis nitidus \| \| Synchita humeralis \| \| Taphrorychus bicolor \| \| Tetratoma ancora \| \| Tetropium castaneum \| \| Tetropium fuscum \| \| Tetrops praeustus \| \| Thamiaraea cinnamomea \| \| Thanasimus femoralis \| \| Thanasimus formicarius \| \| Thymalus limbatus \| \| Tillus elongatus \| \| Tomoxia bucephala \| \| Trachodes hispidus \| \| Trichius fasciatus \| \| Triplax aenea \| \| Triplax rufipes \| \| Triplax russica \| \| Triplax scutellaris \| \| Trypodendron domesticum \| \| Trypodendron lineatum \| \| Trypophloeus bispinulus \| \| Wanachia triguttata \| \| Xyleborus cryptographus \| \| Xylechinus pilosus \| \| Xylita laevigata \| \| Xylophilus corticalis \| \| Xylostiba monilicornis \| \| Zilora ferruginea \| |

**__________________________________________________________________________________**

**Table S3:** Table of measures of biodiversity and variance inflation factors (VIF). To avoid multicollinearity, we systematically dropped the variables with the highest VIF. This was done until only variables with a VIF below 3 remained and these variables were used in our full model.

| **Variable** | **Full VIF** | **Reduced VIF** |
| --- | --- | --- |
| CWM body roundness | 7.471 | Removed |
| CWM body length | 2.582 | 1.763 |
| CWM wing length | 6.420 | 2.504 |
| Prop abundance of predators | 4.112 | 1.873 |
| Species richness of predators | 40.573 | Removed |
| Species richness | 120.913 | Removed |
| Shannon diversity index | 4.848 | 1.951 |
| Abundance | 16.029 | Removed |
| CWM mandibular aspect | 1.523 | 1.201 |
| CWM body width | 4.975 | 1.771 |
| CWM wing load | 6.821 | 2.384 |
| FDis | 2.533 | 2.950 |
| Prop abundance of detritivores | 7.613 | Removed |
| Species richness of detritivores | 37.463 | Removed |
| FRic | 20.764 | Removed |
| FEve | 2.321 | 1.894 |
| Latitude | 2.910 | 2.086 |
| Longitude | 1.820 | 1.605 |

**Table S4:** Results from the model that included predictor variables that were excluded from the full model due to collinearity and were not included in the alternative models in the main text. The model predicted forest management using measures of saproxylic beetle biodiversity captured in flight intercept traps in forests. Forest management (near-natural = 1, managed = 0) was modeled with a generalized linear mixed effect model (GLMM) with binomial distribution and measures of beetle biodiversity as fixed effects, and the sampling project as a random effect.

| **Variable** | **Estimate** | **Std. Error** | **P value** | **AIC** |
| --- | --- | --- | --- | --- |
| Intercept | 3.346 | 6.027 | 0.578 | 382 |
| Species richness of predators | 0.981 | 0.439 | 0.057 |  |
| Abundance | -3.221 | 1.858 | 0.083 |  |
| Prop abundance of detritivores | -0.241 | 0.184 | 0.192 |  |
| Species richness of detritivores | -0.009 | 0.464 | 0.983 |  |
| FRic | -8.083 | 4.969 | 0.103 |  |
